# Effects of Strengthening Exercises on Human Kinetic Chains Based on a Systematic Review

**DOI:** 10.3390/jfmk9010022

**Published:** 2024-01-17

**Authors:** Muhammad Adeel, Bor-Shing Lin, Muhammad Asad Chaudhary, Hung-Chou Chen, Chih-Wei Peng

**Affiliations:** 1 Department of Computer Science and Information Engineering, National Taipei University, New Taipei City 23741, Taiwan; dr.adeel215@gm.ntpu.edu.tw (M.A.); bslin@mail.ntpu.edu.tw (B.-S.L.); 2 School of Biomedical Engineering, College of Biomedical Engineering, Taipei Medical University, 250 Wuxing Street, Taipei 11031, Taiwan; 3 College of Biomedical Engineering, Kaohsiung Medical University, Kaohsiung 80708, Taiwan; asadchaudhary97@gmail.com; 4 Department of Physical Medicine and Rehabilitation, School of Medicine, College of Medicine, Taipei Medical University, Taipei 11031, Taiwan; 10462@s.tmu.edu.tw; 5 Department of Physical Medicine and Rehabilitation, Shuang Ho Hospital, Taipei Medical University, New Taipei City 23561, Taiwan; 6 School of Gerontology and Long-Term Care, College of Nursing, Taipei Medical University, Taipei 11031, Taiwan

**Keywords:** myofascial chains, muscle activation, exercises, maximal voluntary isometric contraction

## Abstract

Kinetic chains (KCs) are primarily affected by the load of different activities that recruit muscles from different regions. We explored the effects of strengthening exercises on KCs through muscle activation. Four databases were searched from 1990 to 2019. The muscles of each KC, their surface electromyography (sEMG), and the exercises conducted were reported. We found 36 studies that presented muscle activation using the percent (%) maximal voluntary isometric contraction (MVIC) or average sEMG for nine KCs in different regions. The % MVIC is presented as the following four categories: low (≤20%), moderate (21~40%), high (41~60%), and very high (>60%). Only four studies mentioned muscle activation in more than three KCs, while the remaining studies reported inconsistent sEMG processing, lacked normalization, and muscle activation in one or two KCs. The roles of stabilizers and the base of support in overhead throwing mobility using balance exercises were examined, and the concentric phase of chin-up and lat pull-down activated the entire KC by recruiting multiple muscles. Also, deep-water running was shown to prevent the risk of falls and enhance balance and stability. In addition, low-load trunk rotations improved the muscles of the back and external oblique activation. Based on this study’s findings, closed-chain exercises activate more groups of muscles in a kinetic chain than open-chain exercises. However, no closed or open chain exercise can activate optimal KCs.

## 1. Introduction

Kinetic chains (KCs) in rehabilitation are defined [1] as “a combination of several successively arranged joints constituting a complex motor unit.” Each bony part of the lower limb, like the foot, leg, thigh, and pelvis, can be seen as a solid link, with the subtalar, ankle, knee, and hip joints serving as connections [2]. In a KC, if two ends of the rigid-link system are fixed so that no movement can occur at either end, applied external forces allow each segment to collect and transfer force to the neighboring segment, creating a chain reaction. Therefore, movement at any joint in the chain causes a consistent movement pattern with all of the other joints [3].

KCs are classified as open or closed based on the weight exerted on the terminal segment. Hence, an open KC (OKC) is “a combination of successively arranged joints in which the distal segment can move freely”, whereas a closed KC (CKC) is “a condition in which the distal segment meets considerable external resistance that restrains its free motion” [1]. It is understood that the type of KC utilized affects muscle recruitment and joint movement patterns [4]. The type and physiology of muscle contractions that underlie joint motion are intimately related. In many daily activities and sports, a common activation sequence uses a CKC, in which the action is initiated from a firm base of support, and the generated force is subsequently transferred to more mobile, distal segments through the links.

The concept of KCs provides a basis for comprehending and successively examining human movement patterns. It is also a helpful tool for executing challenging multi-joint exercises that target an entire KC of the body as a part of conditioning and rehabilitation programs [5,6]. Due to the interlinking of segments, KCs affect the proximal and distal segments’ movements [7,8] by offering several degrees of freedom and different segment positions and motions during sport-specific movements [5,6]. Sometimes, it can be challenging to differentiate between the CKC and OKC. For instance, OKC activities like swimming and cycling place a strain on the distal segment, but that segment is neither stable nor restricted from movement [9].

Myofascial chains (MCs) [10] have recently arisen, which contrasts the KC idea [11,12,13]. MCs describe anatomical and neurophysiological observations that suggest the fascia, a soft tissue with viscoelasticity, features as a functional envelopment linking muscles throughout the entire body, enabling rich sensorimotor communication between body segments [11,12,13]. This notion of neural and musculoskeletal systems acting as interconnected neuromuscular chains goes beyond the current paradigm of understanding musculoskeletal functioning as several isolated single-joint muscular origins and insertions [14]. This myofascial force transmission theory has been supported by several investigations, which contend that intermuscular chains transmit forces to the surrounding tissues through connective tissue envelopes of tendinous attachments between muscles rather than only working as isolated units [15,16].

Recent research on KCs and myofascial principles separately covers these two concepts [6,11,17,18]. These two ideas, however, might be viewed as two sides of the same coin, according to recent studies [8,11]. A study by Licen et al. [19] linked the KC approach with myofascial training in tennis players to maximize rehabilitation and prevention programs, which are primarily intended to reduce the incidence of injuries and positively influence biomechanical patterns of movement, muscle coordination, and muscle force production.

One study explored the effect of muscle activation during isometric movements (active plantar flexion) and found a strong correlation among the muscle activation of the entire kinematic chain, superficial backline (SBL) muscles at the right T12, posterior superior iliac spine (PSIS), and hamstrings. Hence, they reported that muscles do not work independently, so dysfunction in any muscle along any kinematic chain may alter activation patterns and increase myofascial pain (MFP) [20]. Another study presented the effect of changes in the trunk and lower-body position on trapezius activation and observed increased activation during unimodal squats on the contralateral leg compared to traditional seated exercises [21].

Comprehending how muscle activation in various joints interacts to produce biomechanically powerful but also safe movements could enable us to use customized conditioning and rehabilitation strategies to correct poor biomechanics. Because the malfunctioning of a KC indicates a significant injury risk, it is crucial to be aware of the frequent occurrence of pain or injury caused by inefficient biomechanics that result in changed force distributions and power outputs [19].

Strengthening exercises can be performed in a variety of ways, and each of them has an effect. Among them, resistance-band exercises are used for rotator cuff musculature training [22]. A sling exercise with eight variations in scapular retraction in overhead athletes activates the upper and lower trapezius muscles [21]. Body weight exercises like the bench press activate pectoralis major, triceps brachii, biceps brachii, deltoid, and supraspinatus muscles more than push-ups [23]. A modified closed kinetic chain knee extension activates the quadriceps more than an open kinetic chain knee extension [24].

Hence, our current study attempted to identify the evidence from existing research on training the entire KC of the human body through muscle activation. Much of the literature published on the existence of human KCs theoretically and anatomically [13,14] reports the effects of OKC or CKC exercises in a single muscle in a variety of pathological and normal conditions [6,9] including pain, disability, and muscle strength [21,25], but very few studies explore the effects of strengthening exercises on entire KC activation. Likewise, to the best of our knowledge, very little research was found to describe the suspension, sling, or three-degree-of-freedom exercise interventions [26,27,28] needed to train an entire KC and enhance physical or exercise performance. That is why we conducted a systematic review in which KCs of different regions were included, and we hypothesized that the type of exercise would affect kinetic chain activation during strengthening exercises in various regions of the body. So, the main goal of this study was to explore the effects of therapeutic exercises on the entire KCs of the upper limbs, trunk, and lower limbs, and the secondary goal was to highlight which exercises activated the entire KC in better ways to enhance physical performance compared to the single muscle activation of a KC.

## 2. Materials and Methods

### 2.1. Reporting and Registration

The Preferred Reporting Items for Systematic Reviews and Meta-Analysis (PRISMA) statement was fulfilled [29,30]. This review was registered before the initiation of the study with PROSPERO (registration no.: CRD42020142495).

### 2.2. The Literature Search Strategy

**Figure 1:**
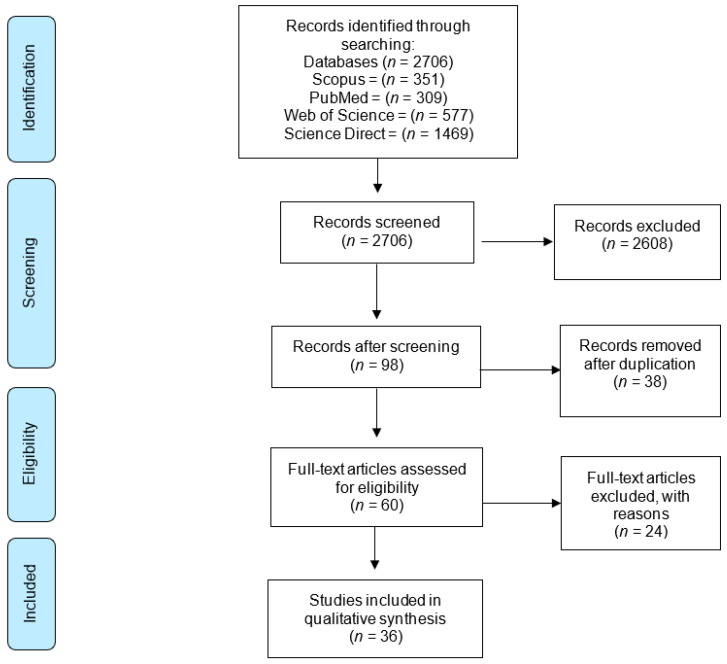
PRISMA diagram.

A search of the literature was performed from 1 January 1990 to 21 July 2019 and the first evidence of the effect of exercises on the functioning and performance of the kinetic chain was found in 1990. (Figure 1) We searched the four databases of Scopus, PubMed, Web of Science, and ScienceDirect using the following key terms: (1) ((“myofascial chain” OR “kinetic chain” OR “muscular sling” OR “anatomy train”) AND (exercise AND therapy OR strength AND training)), (2) ((“myofascial chain” OR “kinetic chain” OR “muscular sling” OR “anatomy train”) AND (“exercise therapy” OR “strength training”)), and (3) ((“myofascial chain” OR “kinetic chain” OR “muscular sling” OR “anatomy train”) AND (“exercise therapy” OR “strength training” OR ‘‘open chain’’ OR ‘‘close chain’’)).

### 2.3. Study Selection

The articles were selected if they (1) were written in English, (2) had healthy participants of both genders aged 15~50 years, (3) had participants with no history of a previous systemic illness, surgery, or musculoskeletal impairment, (4) were experimental studies including observational, case–control, cross-sectional, case report, quasi-experimental, and randomized controlled trials (RCTs), (5) were studies that included KCs of different regions like the upper limbs, trunk, or lower limbs, (6) were studies that mentioned the muscles included for exercise therapy, (7) examined a KC that included more than two muscles and fascia, (8) included exercise interventions in strength training that included suspension or sling and three degrees of freedom exercises [26,27,28], with such exercises being performed with some exercise gadgets like bands, cords, or exercise machines, and (9) were studies that specifically focused on outcomes related to body functions (weight maintenance and movement-related functions and activities) and participation (such as motor skills, carrying out tasks, or mobility and walking indices). Studies were excluded if they (1) reported only intervention effects (exercise therapy effects without considering human KCs, groups of muscles or describing biomechanical performance through kinematic or kinetic variables), (2) mentioned the exercise effect on muscle activity without mentioning the included muscles, (3) were anatomical studies confirming myofascial connections, (4) were studies other than those with experimental designs like qualitative surveys or theoretical studies, and (5) included exercises performed using assistive gadgets like orthosis or exercise therapy that focused on the effects of posture.

### 2.4. Risk of Bias Assessment

A modified version of the Quality Assessment Tool for Quantitative Studies [31] was employed to assess the quality of the articles. Two reviewers independently assessed the methodological quality of the articles according to the criteria established earlier [32]. If independent quality scores were within a 10% difference of each other, the average score of the two reviewers was used. If the scores differed by more than 10% between the reviewers, quality scoring was discussed, and if required, a third independent reviewer was consulted [33].

### 2.5. Quality of Reporting Assessment

The Effective Public Health Practice Project Quality Assessment Tool (EPHPP) was used to assess study designs like RCTs, before-and-after, and case–control studies. It is an effective tool for use in systematic reviews of effectiveness [34] with content and construct validities [35,36]. The EPHPP evaluates the following six domains: (1) selection bias, (2) study design, (3) confounders, (4) blinding, (5) data collection method, and (6) withdrawals/dropouts (Table 2). Each domain was scored as strong (3 points), moderate (2 points), or weak (1 point), and scores were averaged to obtain a total score or overall quality. Depending on the overall score, studies were given a quality rating of weak (1.00–1.50), moderate (1.51–2.50), or strong (2.51–3.00) [31].

### 2.6. Data Extraction and Analysis

The characteristics of participants (the number of participants, age, gender, weight, and height) [32], characteristics of the involved KCs (region, muscle, joints), outcome measurements (muscle activation, muscle strength, physical functioning, activity performance), exercise interventions (exercise type, intensity, load, and gadgets), and an improvement in performance through peak muscle activation (surface electromyography (sEMG)) or percent (%) maximal voluntary isometric contraction (MVIC) were extracted. The normalized % MVIC was used to report muscle activation (sEMG) as low (≤20% MVIC), moderate (21~40%), high (41~60%), and very high (>60%) [37,38,39,40,41]. The data of some studies were extracted from figures (if not available in tabulated form) using WebPlotDigitizer (accessed at https://automeris.io/WebPlotDigitizer/) (accessed on 5 May 2023). One reviewer extracted the data, which were then verified independently by a second reviewer. Data were tabulated in Microsoft^®^ Office Excel (LTSC MSO (16.0.14332.20611)), and descriptive statistics were presented, including the means and SDs. Study outcome measures were (1) muscle strength through a repetition maximum (RM), manual muscle testing (MMT), and sEMG, and (2) physical functioning or activity performance through the change in activity levels after exercises assessed using a subjective tool.

## 3. Results

### 3.1. Quality of Reporting Assessment

Out of 36 studies, 6 showed moderate quality, while the remaining studies were rated as weak. Almost all studies had strong selection bias and data collection methods, but study design and blinding were weak because of smaller sample sizes, a lack of study groups, and a lack of a randomized controlled design. None of them reported confounders, and this category was rated as moderate. In addition, withdrawals and dropouts were also in the moderate category (Table 1).

### 3.2. Participant Characteristics

The sample sizes of the included studies ranged from 5 to 47 participants of both genders. Out of 36 studies, 19 recruited both genders, 11 had only males, 4 had only females, and 2 studies did not mention gender. Ages ranged from 11 to 43 years, body weights ranged from 36 to 107 kg, and heights ranged from 143 to 185 cm (Table 2).

### 3.3. Studies Features

Out of the 36 studies, 17 were related to an upper extremity (UE), 11 to a lower extremity (LE), 2 to the trunk (Tr), 3 to UE and LE, 1 to UE and Tr, and 2 to LE and Tr.

#### 3.3.1. Muscles

The UE included shoulder and scapular muscles, the LE included hip and knee muscles, and the Tr included abdominal and back muscles.

#### 3.3.2. Exercises

In total, 21 out of the 36 studies tested CKC exercises with a stable or unstable base of support, isometric or isotonic muscle contractions, and only 5 studies researched OKC exercises. The remaining 10 studies did not mention whether they employed CKC or OKC exercises despite their included exercises focusing on a range of motion (ROM), balance, mobility, and isometrics.

### 3.4. Effects of Exercise Therapy on Human KCs

Overall, nine KCs were considered, which were the elbow, shoulder, scapula, abdomen, back of spine, pelvis, hip, knee, and ankle, based on muscle involvement. Different types of OKC, CKC, ROM, and balance exercises affected the muscle activation of a specific KC of the UE, LE, Tr, or a combination except for [22,52,59,63], which reported more than three KCs of different regions. The region-specific KCs are reported in Table 3.

### 3.5. Exercise Type and Muscle Activation (sEMG)

The muscle activity reported through sEMG or integrated EMG (iEMG) in % MVIC normalized values is considered to present the effect of exercise therapy on KC muscles in this review. Of the 36 studies included, we did not compare the muscle activation of some studies due to the lack of a normalization process and presentation of average sEMG in microvolts (µV) or iEMG in volts (V) by (1) [44], (2) [47], (3) [69], and (4) [73]. DeCarlo et al. [73] did not report muscle activation, but rather, they interpreted outcomes based on ROM and angular velocity. There was an exception for this paper [44] because it was retracted by the journal. Therefore, we described the muscle activation of 32 studies according to the reported KCs. (Table 4).

### 3.6. Kinetic Chain Muscle Activation (sEMG)

Of the 32 studies, only 4 of them [22,52,59,63] included more than three KCs of different regions. Therefore, we describe and discuss the KC muscle activation of these studies in detail in the Discussion section.

## 4. Discussion

The current review is the first study to summarize the available literature on KC activation by exploring the effects of strengthening exercises. Based on these studies, the roles of stabilizers and the base of support in overhead throwing mobility using balance exercises were examined, and the concentric phase of chin-up and lat pull-down exercises were found to activate the entire KC by recruiting multiple muscles of the body. Also, deep-water running prevents the risk of falls and enhances balance and stability in elderly people. In addition, low-load trunk rotations can improve the muscles of the back and external oblique activation in athletes and healthy populations after an injury.

In all 36 studies, serratus anterior (SA), upper trapezius (UT), anterior deltoid (AD), lower trapezius (LT), and pectoralis major (PM) from the upper extremity, biceps femoris (BF), rectus femoris (RF), vastus lateralis (VL), gluteus maximus (GMax), and gluteus medius (GMed) from the lower extremity, and erector spinae (ES), rectus abdominus (RA), and external oblique (EO) from the trunk were the most often studied muscles. Muscles in a KC are sequentially activated, some of which are highly active, while others are less active. Hence, this research area can help determine the best exercise intervention to challenge or train an entire KC. Applied research on this topic is limited, which may pose greater benefits to prevent injuries and enhance physical performance through optimal muscle activation in a KC.

A study [22] reported on muscle activation in exercises with the following involved muscles: (1) airplane internal rotation: UT, LT, and BF; (2) airplane external rotation: LT, GMed, and BF; (3) lunge: UT, LT, SA, and LD; (4) get-up exercise: SA; (5) single-leg balance: UT; (6) I-band: UT, LT, and SA; (7) T-band: UT and LT; and (8) Y-band: LT was moderately active. This study included dominant-side UT, LT, SA, and LD and bilateral GMed and BF muscles with a focus on the posterior chain. In overhead throwing, stabilizers transfer kinetic energy from the lower extremity through the pelvis, trunk, and scapula onto the shoulder [74]. Therefore, stabilizers should be included in rehabilitation to prevent injury and maximize the throwing performance [22]. KC exercises (airplane ER and IR) moderately activate pelvic muscles along with the scapular musculature compared to band exercises due to the greater challenge of these muscles for single-leg balance and lumbopelvic stability. Another KC exercise, the lunge, also greatly activates scapular stabilizers (UT, LT, SA, and LD) [74]. Thus, challenging the entire KC can target not only the prime movers but also stabilizers for efficient movement performance.

Another study [52] reported that during the concentric phase, the muscle activation of the biceps brachii, latissimus dorsi, and ES were high during chin-ups, while those of LD and RA appeared high during the lat pull-down. During the eccentric phase, the activation of LD and BB was high during chin-ups, while lat pull-down showed higher activation for LD. The greater activation of BB and ES during chin-ups was due to an unstable position compared to lat pull-down, which was performed in a stable position [52]. In chin-ups, the LE can move freely in a horizontal plane, resulting in the displacement of the body’s center of gravity from the vertical alignment of holding the bar [75]. The PM, TB, and RA muscles were not affected by differences in the stability condition, as the same results were shown by previous studies [62,76,77,78]. The extent of the instability experienced by each muscle was proposed to be due to the anatomical orientation of the muscles [77]. Due to greater stability and movement demands at the glenohumeral and elbow joints, BB is highly active during chin-ups and also a biarticular muscle compared to the LD, which is a prime mover but a monoarticular muscle [77]. The higher activation of the ES during concentric chin-ups and the RA during eccentric lat pull-downs implied the engagement of core muscles to stabilize the body to a greater extent during these exercises, as mentioned by other studies [79,80,81]. However, this study performed only a sagittal plane analysis of chin-ups and lat pull-downs, but these exercises can be utilized for strengthening the upper back and arm muscles and are performed by gymnasts and rock climbers, which require the greater stability of the body while hanging from their hands [52].

One study [59] reported that the GMax, GMed, and ES had higher activation during deep water running (DWR) at slow, moderate, and fast speeds, while the AL, EO, and RA muscle activation levels were lower. The increased muscle activity during DWR and fast-speed water walking (WW) was due to an increased hip ROM, trunk forward inclination, and an unstable floating condition. Due to the large hip joint ROM, pelvic stabilization in an unstable condition was more challenging for the GMed and AL during DWR [59]. An increased hip extension caused the higher activation of the GMax and BF during DWR than land walking (LW). Also, fast-speed DWR reduces the cycle time and results in higher GMax activity to overcome water propulsion resistance, which increases as the second power of the speed [82]. To control hip extension movement and backward pelvic rotation, the RA and EO are highly active, while the ES tends to overcome water resistance [59]. This study involved trunk and hip adductor and abductor muscles to control the pelvic and trunk stability and balance in the elderly rather than the young [83,84]. In rehabilitation, water exercises prevent the risk of falls, unlike walking on land. So, DWR can be applied for coxarthropathy and lower-extremity rehabilitation after injury [59].

A study by Stevens et al. [63] mentioned that all of the included muscles reach a strength level at a 30% load except for the RA and IO, which may be trained in the flexion position rather than in the rotation. This intensity poses minimal tissue loads due to low resistance and a controlled neutral lumbar spine position [85]. The IO, EO, and LD are connected by the thoracolumbar fascia (TLF), and the MF and ICLT have hydraulic amplifier effects on layers of the TLF [86]. Due to these connections, the TLF maintains tension in these local and global trunk muscles [87]. Hence, a low-load-seated rotation can train the back muscles and EO during early rehabilitation after an injury [63].

The limitations include the following: (1) Only a few research studies were found with experimental designs to study therapeutic exercise effects on entire KCs. (2) There is a lack of focus on a single movement or family of movements to identify which muscles are involved in that specific kinetic chain and to what extent each one is involved in the movement. (3) There is a lack of movement-specific and particular body area analyses of the entire kinetic chain. (4) Due to inconsistent muscle activation data methods, a different group of involved muscles and varying kinds of exercises, such as a meta-analysis, were unable to be conducted.

Future work may include the following: (1) A good experimental design, particularly a randomized controlled trial for future research. (2) The same kind of studies should be conducted on older adults and people with diseases for an individualized exercise program. (3) Most of the research was focused on one or two muscle groups. So, in the future, studies should focus on the entire kinetic chain of different regions to examine the response during exercise regimens to recruit upper extremity, lower extremity, and trunk muscles. (4) About 60% of the included studies reported closed kinetic chain exercises, and only 14% of them reported open kinetic chain exercises. So, well-designed studies with mixed types of exercises that have better implications for athletes, healthy populations, and patients are required. (5) Overall, by increasing the load, muscle activation increases, but how it affects the entire kinetic chain is a missing area of research. (6) Most of these studies worked on UE and LE muscles like the SA, UT, ADelt, LT, PM, BF, RF, VL, GMax, and GT, but much less work was found on the LD, TB, BB, ES, GMed, TA, SOL, and peronei, etc., which should be considered in future research. (7) The search string should be tested with a simpler term to refer to kinetic or myofascial chains as “muscle chains” or “intermuscular coordination” in future research.

## 5. Conclusions

Kinetic chain exercises activate single or multiple groups of muscles. Based on the study findings, closed-chain exercises activate more groups of muscles in a kinetic chain than open-chain exercises. However, no closed or open-chain exercise can activate the optimal muscles of different regions simultaneously. However, a mixed approach using closed and open-chain exercises together can target more groups of muscles in an entire kinetic chain.

## Figures and Tables

**Table 1:** Quality Assessment Tool for Quantitative Studies (EPHPP) (*N* = 36).

*N*	Selection Bias	Study Design	Confounders	Blinding	Data Collection Method	Withdrawals and Dropouts	Overall Quality
[22]	strong	weak	moderate	weak	strong	moderate	weak
[42]	strong	weak	moderate	weak	strong	moderate	weak
[43]	strong	weak	moderate	weak	strong	moderate	weak
[44]	strong	weak	moderate	moderate	strong	moderate	moderate
[45]	strong	weak	moderate	weak	strong	moderate	weak
[46]	strong	weak	moderate	weak	strong	moderate	weak
[47]	strong	weak	moderate	weak	strong	moderate	weak
[48]	strong	weak	moderate	weak	strong	moderate	weak
[49]	strong	weak	moderate	weak	strong	moderate	weak
[50]	strong	weak	moderate	weak	strong	moderate	weak
[51]	strong	weak	moderate	moderate	strong	moderate	moderate
[52]	strong	weak	moderate	weak	strong	moderate	weak
[53]	strong	weak	moderate	weak	strong	moderate	weak
[54]	strong	weak	moderate	weak	strong	moderate	weak
[55]	strong	weak	moderate	weak	strong	moderate	weak
[23]	strong	weak	moderate	weak	strong	moderate	weak
[56]	strong	weak	moderate	weak	strong	moderate	weak
[57]	strong	weak	moderate	weak	strong	moderate	weak
[58]	strong	weak	moderate	weak	strong	moderate	weak
[59]	strong	weak	moderate	weak	strong	moderate	weak
[60]	strong	weak	moderate	weak	strong	moderate	weak
[61]	moderate	moderate	moderate	weak	strong	strong	moderate
[62]	strong	weak	moderate	weak	strong	moderate	weak
[63]	strong	weak	moderate	weak	strong	moderate	weak
[37]	strong	weak	strong	weak	strong	moderate	weak
[64]	strong	weak	moderate	moderate	strong	moderate	moderate
[65]	strong	weak	strong	weak	strong	moderate	weak
[66]	strong	weak	moderate	weak	strong	moderate	weak
[67]	strong	weak	moderate	weak	strong	moderate	weak
[68]	strong	weak	moderate	moderate	strong	moderate	moderate
[69]	strong	weak	moderate	weak	strong	moderate	weak
[24]	strong	weak	moderate	moderate	strong	moderate	moderate
[70]	strong	weak	moderate	weak	strong	moderate	weak
[71]	strong	weak	moderate	weak	strong	moderate	weak
[72]	strong	weak	moderate	weak	strong	moderate	weak
[73]	strong	weak	moderate	weak	strong	moderate	weak

*N*, number of included studies. quality assessment of included studies as three categories: strong (no weak ratings), moderate (one weak rating), and weak (two or more weak ratings). overall quality, the qualitative assessment tool for the quantitative studies scale was used for the quality assessment of included papers. This scale has a total of six categories, including (A) selection bias, (B) study design, (C) confounders, (D) blinding, (E) data collection method, and (F) withdrawals and dropouts.

**Table 2:** Basic demographics of participants (*N* = 36).

*N*	*n*	Gender	Age (Years)	Weight (kg)	Height (cm)
[22]	26	Both	22.9 ± 3.4	74.2 ± 16.3	172.2 ± 8.6
[42]	15	M	20.5 ± 2.2	63.8 ± 6.0	174.5 ± 5.3
[43]	20	--	11.3 ± 1.0	47.5 ± 11.3	152.4 ± 9.0
[44]	21	M = 19/F = 10	21.5 ± 4.7	60.1 ± 11.4	164.2 ± 6.2
[45]	21	M = 10/F = 11	22.8 ± 1.4	--	--
[46]	13	F	28.9 ± 5.1	58.2 ± 6.4	164.0 ± 6.3
[47]	14	M	14.1 ± 0.8	71.9 ± 13.6	171.0 ± 7.0
[48]	30	M = 13/F = 17	22.3 ± 0.9	61.6 ± 9.9	170.9 ± 8.4
[49]	47	M = 26/F = 21	22.0 ± 4.3	69.0 ± 8.6	176.0 ± 0.1
[50]	30	Both	23.5 ± 1.3	76.6 ± 16.9	174.4 ± 11.0
[51]	42	M = 18/F = 24	43.0 ± 11.0	71.0 ± 10.0	171.5 ± 6.5
[52]	9	M	26.0 ± 9.0	82.6 ± 12.0	179.0 ± 6.0
[53]	30	M = 10/F = 20	20.1 ± 1.3	75.0 ± 9.3	175.6 ± 4.7
[54]	20	M	22.8 ± 3.1	68.7 ± 7.9	175.0 ± 5.0
[55]	19	M = 11/F = 8	23.2 ± 2.3	61.3 ± 9.7	168.2 ± 7.3
[23]	15	M	19.8 ± 1.4	69.3 ± 5.0	176.8 ± 4.2
[56]	10	M = 3/F = 7	25.0 ± 5.0	78.0 ± 15.0	171.0 ± 7.0
[57]	8	M	--	--	--
[58]	32	M = 16/F = 16	22.9 ± 2.4	65.6 ± 8.1	173.0 ± 9.0
[59]	9	M	25.1 ± 2.3	65.7 ± 4.1	168.5 ± 4.2
[60]	20	M = 11/F = 9	20.9 ± 1.9	73.3 ± 10.6	--
[61]	34	M = 13/F = 15	20.9 ± 2.7	71.7 ± 11.0	174.4 ± 8.1
[62]	12	M	23.0 ± 7.0	72.0 ± 15.0	172.0 ± 9.0
[63]	30	--	20.5 ± 1.7	64.3 ± 10.0	172.7 ± 8.7
[37]	5	M	24~32	--	--
[64]	6	F	30.3 ± 7.7	51.0 ± 3.4	159.0 ± 4.0
[65]	5	M	24~32	--	--
[66]	15	M = 7/F = 8	22.1 ± 0.7	64.7 ± 6.4	170.2 ± 6.6
[67]	20	M = 10/F = 10	25.0 ± 5.3	72.2 ± 9.7	164.1 ± 6.8
[68]	12	Both	21.7 ± 2.8	73.7 ± 17.1	168.6 ± 23.8
[69]	18	M = 10/F = 8	21.0 ± 8.0	69.7 ± 13.0	173.0 ± 11.0
[24]	15	F	18~26	57.7 ± 4.2	--
[70]	20	F	31.3 ± 6.9	58.1 ± 8.7	160.9 ± 4.1
[71]	41	M = 20/F = 21	21~39	51.4~115.9	157.5~195.6
[72]	10	M	30.0 ± 6.0	93.0 ± 14.0	177.0 ± 9.0
[73]	10	M = 5/F = 5	26.1 ± 0.9	--	--

*N*, number of included studies; *n*, number of participants; M, male; F, female; kg, kilogram; cm, centimeter.

**Table 3:** The region and kinetic chains of the included studies (*N* = 36).

Region	Kinetic Chains	Included Studies
UE (*n* = 17)	shoulder, scapula	[42,44,45,49,53,56,57,58,61,67,68]
	elbow, shoulder, scapula	[23,37,48,54,62,65]
LE (*n* = 11)	hip, knee	[46,47,60,66]
	knee, ankle	[64,69,72]
	hip, knee, ankle	[24,70,71,73]
Tr (*n* = 2)	abdomen, pelvis	[55]
	shoulder, abdomen, back of spine, pelvis	[63]
UE and LE (*n* = 3)	shoulder, scapula, hip, knee	[22]
	shoulder, scapula, hip	[43,50]
UE and Tr (*n* = 1)	elbow, shoulder, scapula, abdomen, back of spine, pelvis	[52]
LE and Tr (*n* = 2)	back of the spine, hip, knee	[51]
	abdomen, back of spine, pelvis, hip	[59]

*N*, number of studies; UE, upper extremity; LE, lower extremity; Tr, trunk; KC, kinetic chains are reported according to the region and muscle included by each study.

**Table 4:** Study features and outcome measures (*N* = 36).

Article	Region	Muscles	Exercise	Parameters	Muscle Activation
[42]	UE	UT, LT, SA, ADelt, IS	OKC: robbery exercise while seated and standing: shoulder position; (1) 20° abduction [W], (2) 90° abduction [90/90]	12 sets, 5 reps/set, 0%, 3%, 7% BW, 45 bpm **% MVIC sEMG**	**Seated 90/90: UT:** 85.4 (26.7), **IS:** 82.3 (15.0), **SA:** 71.1 (25.9), **LT:** 66.1 (17.9), **ADelt:** 55.3 (27.5)
[44]	UE	UT, IS, PM, MDelt	OKC: (1) 150° shoulder flexion scapular plane (SP); elbow joint extended, wrist 85° dorsiflexion: 5% body weight (BW) force, (2) same as condition 1: 10% BW force, (3) 120° shoulder flex SP: 5% BW force, (4) same as condition 3: 10% BW force, (5) 90° flexion SP: 5% BW force, (6) same as condition 5: 10% BW force	conditions 1~6 compared **iEMG (µV)**	**Condition 2: MDelt:** 1121.3 (603.2) **Condition 1: IS:** 527.2 (259.3) **Condition 1: UT:** 422.9 (153.0)
[45]	UE	SA, IS, ADelt, PM, LD	CKC: Exercise 1~7: press up 7 static variations	3 reps with 10 s hold **% MVIC sEMG**	**Exercise 6: SA:** 133.9 (69.5), **IS:** 77.0 (50.1), **ADelt:** 47.6 (23.9), **LD:** 47.4 (38.0), **PM:** 20.9 (13.4)
[49]	UE	UT, MT, LT, SA, PM, ADelt, PDelt, LD	CKC: (1) half push-up, (2) knee push-up, (3) knee-prone bridging plus, (4) pull-up without vs. with red cord sling (RS)	5 reps, 3 s concentric and 3 s eccentric, 60 bpm **normalized sEMG**	**RS: Half push-up: PM:** 94.0 (62.9), **PDelt:** 29.9 (24.0). **Pull-up: LD:** 83.5 (60.1) **No RS: Knee-prone bridging plus: SA:** 57.0 (27.2), **ADelt:** 71.3 (52.3), **Pull-up: MT:** 68.8 (29.4), **LT**: 69.4 (33.7).
[53]	UE	SA, UT, MT, LT	CKC: (1) cuff link (CL), (2) push-up (PU), (3) supine pull-up (SP)	5 trials, 2 s/trial % **MVIC sEMG**	**SP: MT:** 62.5 (86.5), **UT:** 61.4 (91.3), **LT:** 60.2 (95.6). **PU: SA:** 50.2 (69.0) **No overhead: LT:** 36.5 (33.1), **UT:** 34.6 (36.3), **Overhead: SA:** 40.5 (22.1)
[56]	UE	ADelt, UT, LT, SA, SS, IS	(1) supine elevation with the opposite hand, (2) forward bow, (3) washcloth press-up hands close, (4) towel slide, (5) scapular protraction on ball, (6) washcloth press-up hands farther apart, (7) supine press-up, (8) wedge press-up, (9) ipsilateral (ips) step-up with ball, (10) ips step-up, no ball, (11) ips shoulder flexion, (12) standing press-up	8 reps, 12 exercises **% MVIC sEMG**	**Standing press-up:** **ADelt:** 31.0 (11.0), **SS:** 29.0 (18.0), **UT:** 24.0 (8.0), **SA:** 29.0 (13.0). **Ips step-up with ball:** **UT:** 24.0 (8.0)
[57]	UE	IS, PM, SA, MT	scapular retraction supinated row	20 reps, 50% isometric maximum **% MVIC sEMG**	**Maximal external rotation:** **30~0°: MT:** 108.0, **SA:** 92.0
[58]	UE	UT, MT, LT, SA	CKC: (1) standard knee push-up plus (KPP), (2) KPP heterolateral leg extension (ext), (3) KPP homolateral leg ext, (4) KPP wobble board, (5) KPP heterolateral leg ext and wobble board, (6) KPP homolateral leg ext and wobble board, (7) one-handed KPP	5 reps, 60 bpm/rep **% MVIC sEMG**	**Exercise 3: SA:** 44.2 (18.7) **Exercise 2: LT:** 20.1 (10.1)
[61]	UE	SA, MT, LT	CKC: (1) cuff link, (2) standard push-up	10 revs or reps/2 s, 60 bpm **% MVIC sEMG**	**Cuff link: SA:** 74.4 (114.4). **Push-up: LT:** 36.2 (55.2), **MT:** 27.0 (47.2)
[67]	UE	UT, ADelt, SA, PM	(1) non-weight bearing, (2) partial weight bearing, (3) full weight bearing	3 trials, 5 revolutions **% MVIC sEMG**	**Full weight bearing: SA:** 81.4 (96.6), **PM:** 35.4 (27.4), **ADelt:** 22.7 (19.1)
[68]	UE	PM, ADelt, PDelt, IS, SS	(1) unsupported, (2) supported–vertical (short lever arm) and diagonal (long lever arm) exercises	**% MVIC sEMG**	**Unsupported: Diagonal: SS:** 21.6 (10.5)
[48]	UE	UT, LT, SA, BB, TMaj, PDelt	CKC: (1~3) stable base, (4~6) without a stable base	each exercise performed for 10 s **% MVIC sEMG**	**Position 2: SA:** 112.0 (2.0), **LT:** 84.0 (2.0), **TMaj:** 74.0 (2.0), **PDelt:** 67.0 (2.0), **Position 6: UT:** 94.0 (2.0), **Position 3: BB:** 65.0 (2.0)
[54]	UE	BB, TB, ADelt, PDelt, UT, SA, PM	CKC: (1) wall press, (2) bench press	3 trials, 6 s hold at 80% load **% MVIC sEMG**	**Bench press:** **SA:** 36.6 (14.3), **TB:** 33.5 (13.4)
[23]	UE	PM, TB, BB, SS, ADelt, MDelt, PDelt	CKC: (1) push-up, (2) bench press	12 s **% MVIC sEMG**	**Bench press: PM:** 99.8 (110.6), **TB:** 93.1 (103.0), **MDelt:** 64.3 (74.2), **BB:** 42.3 (52.4), **SS:** 38.0 (48.5). **Push-up: ADelt:** 60.3 (70.5)
[62]	UE	BB, ADelt, PM, UT, SA	CKC: dominant side exercise: (1) wall press(stable base), (2) wall press(medicine ball), (3) push up(stable base), (4) push up(medicine ball), (5) bench press(stable base), (6) bench press(medicine ball)	3 trials, 4 s **% MVIC sEMG**	**Wall press on medicine ball: UT:** 90.0 (160.0). **Push-up standard: SA:** 77.3 (123.0), **PM:** 49.5 (89.0). **Push-up on medicine ball: ADelt:** 67.7 (90.0)
[37]	UE	SS, IS, USSC, ADelt, MDelt, PDelt, UT, LT, MT, SA, BB	CKC: (1~3) crossbody rotation (CBR) high, mid, low, (4) overhead reach (OHR), (5) ipsilateral floor touch (IFT) L: low, N: no stepping, S: stepping	10 reps, 5 exercises statically (no stepping) and 5 dynamically (with stepping) **% MVIC sEMG**	**CBRLN: USSC:** 68.3 (150.8) **CBRLS: SA:** 200.7 (390.5) **OHRS: UT:** 28.0 (44.4), **SS:** 23.0 (48.8), **MT:** 20.7 (32.6) **IFTS: LT:** 28.6 (52.3)
[65]	UE	SS, IS, USSC, ADelt, MDelt, PDelt, UT, MT, LT, SA, BB	(1) scapular clock counterclockwise (SCCCW), (2) scapular clock clockwise (SCCW), (3) scapular depression (SCD), (4) scapular elevation (SCE), (5) scapular protraction (SCP), (6) scapular retraction (SCR)	1 set, 10 reps **% MVIC sEMG**	**SCCCW: UT:** 91.0 (147.0) **SCCW: USSC:** 61.8 (114.8), **SS:** 53.1 (86.1), **ADelt:** 28.0 (68.5) SCD: **SA:** 46.5 (87.3) **SCR: MT:** 93.3 (130.2), **LT:** 71.8 (102.8)
[46]	LE	GMax, BF, VL	CKC: (1) back squat, (2) barbell hip thrust	10 reps, 10 RM (dynamic) and 3 s isohold (static) **% MVIC sEMG**	**Static Hip thrust: LGMax:** 115.7 (47.4), **UGMax:** 87.1 (79.4), **BF:** 42.5 (29.6). **Squat: VL:** 133.7 (107.6)
[47]	LE	RF, GMax, BF	CKC: bilateral hip extension on glute machine	3 explosive reps, 1.5 s/rep **sEMG (µV)**	**Right side muscles: GMax:** 70.1 (78.9), **RF:** 66.6 (74.6), **BF:** 47.6 (58.6)
[60]	LE	BF, RF, VL	CKC: (1) leg extension, (2) squat, (3) deadlift, (4) lunge, (5) step up	2 full ROM reps-6 RM load **% MVIC sEMG**	**Deadlift: BF:** 54.7 (75.5). **Leg extension: RF:** 86.6 (101.7). **Lunge and Squat: VL:** 93.3 (106.6) and 90.0 (109.0)
[66]	LE	VM, VL, BF, SM	jumping	3 trials **% MVIC sEMG**	**15~55° knee ROM: Females: VL:** 223.3 (338.2), **VM:** 213.5 (286.2). **Males: BF:** 50.1 (74.3). **Both: SM:** 36.9 (68.1)
[64]	LE	RF, BF, GT	CKC: leg press in two postures: supine and trunk upright	two 5 s MVCs, 6 knee angles 15°, 30°, 45°, 60°, 75°, and 90° **% MVIC iEMG**	**Supine: RF:** 90°: 45.3 (7.3), **GT: 30°:** 45.0 (4.7) **Trunk upright: GT**: 30°: 58.0 (4.5), **RF: 90°:** 45.0 (7.6), **BF: 15°:** 26.5 (2.3)
[69]	LE	TA, PL, SOL, LGT	(1) single-leg stance (SLS), (2) side-stepping (SSt), (3) high knees (HK)–with sandals and foam surface, (4) Tband kicks–sagittal and frontal planes	3 trials-SLS (12 s), SSt and HK: 10 cycles (52 bpm) T-band kicks: 20 kicks (112 bpm) **mean sEMG (V)**	**SLS: SOL:** 0.6 (0.9), **LG:** 0.5 (0.6) **HK:** TA: 1.0 (1.4), **PL:** 1.0 (1.4) **SLS vs. Tband: SLS: TA:** 1.0 (0.4), **PL:** 1.0 (0.4), **SOL:** 0.6 (0.3), **LG:** 0.5 (0.1)
[72]	LE	VM, VL, RF, BF, SM, ST, GT	CKC: (1) leg press, (2) squat, (3) knee extension	4 reps, 12 RM load **% MVIC sEMG**	**Squat: 88~102°: VM:** 61.0 (12.0), **VL:** 54.0 (8.0), **RF:** 48.0 (18.0), **60~74°: BF:** 36.0 (13.0), **SM-ST:** 33.0 (12.0). **Knee extension: 11~26°: VL:** 54.0 (8.0), **RF:** 48.0 (18.0)
[24]	LE	VL, RF, SM, ST, BF, GT, TFL, GMax	OKC and CKC: isometric knee extension	1 set 3 MVIC trials, 3 s hold **normalized iEMG**	**CKC: VM:** 0.3 (0.4) **OKC: RF:** 0.3 (0.3)
[70]	LE	GMax, Hams, VL, SOL	CKC: (1) wall slide, (2) squat in four positions: IP:FF, H1P:I-L, SCAP:FF, SCAPI-L.	5 reps, 80 bpm (4 count/rep) **% MVIC sEMG**	**Squat: GMax:** 30.5 (9.3), **Hams:** 26.6 (9.9). **Wall press: SOL:** 91.8 (24.3), **VL:** 27.0 (13.2)
[71]	LE	GMax, Hams, VM, RF, VL, GT	CKC: unloaded squat	3 trials, 4 reps, 50 bpm (3.6 s/rep) **% MVIC sEMG**	**Concentric: 90~60°: VM:** 67.6 (65.2), **VL:** 62.7 (77.3) **Eccentric: RF:** 48.5 (38.7) **Isometric: 90°: RF:** 48.3 (44.8)
[73]	LE	GMax, QF, Hams, TA, MGT	(1) CKC–Stairmaster machine (SM) (2) OKC–flexion-extension-Cybex	**SM:** 1 set and 7 loads: low, 6, 8, 10, 12, 14, 16 (2~3 min). **Cybex:** 3 reps-60° and 180°, 20 reps-240° (2 s/rep)	--
[55]	Tr	RA, EO, TrA/IO	single leg hold (SLH)–floor vs. foam roller	5 s hold **% MVIC sEMG**	**Foam roller: EO:** 39.7 (19.6), **TrA/IO:** 31.8 (16.9), **RA:** 23.4 (10.7)
[63]	Tr	IO, EO, RA, MF, ICLT, LTh, LD	seated axial submaximal dynamic rotations	5 reps, 5 s at 30%, 50%, 70% load **% MVIC sEMG**	**Away from midline:** **Ipsilateral: ICLT: 70% load:** 131.5, **50%**: 118.4, **30%**: 88.5 **Contralateral: MF: 70%:** 101.3, **50%**: 91.4, **30%**: 73.8
[22]	UE, LE	GMed, BF, LD, LT, UT, SA	(1) airplane ER, (2) airplane IR, (3) lunge, (4) get-up exercise, (5) single-leg balance, (6~8) traditional resistance exercise: I, T, Y	3 trials, 2 s/rep **% MVIC sEMG**	**Airplane IR: BF:** 29.9 (11.8) **Airplane ER: GM:** 20.3 (11.7)
[43]	UE, LE	GMed, LT, UT, SA	OKC: maximal throw effort 4-seam fastballs for strikes: fastest throw selected for analysis	phase 1: foot contact phase 2: ball release ER phase 3: ball release IR **% MVIC sEMG**	**Phase 2: RGMed:** 66.8 (18.6), **UT:** 45.8 (21.6), **SA:** 31.5 (14.3) **Phase 3: UT:** 45.5 (21.3), **SA:** 36.8 (19.1), **RGMed:** 31.9 (10.6)
[50]	UE, LE	GMax, LT, UT, IS, ADelt	(1) static overhead (OH), (2–3) static and dynamic physioball (PB), (4) shoulder dump, (5–6) ipsilateral (ips) and contralateral (cont) leg external rotation (ER), (7) static abduction (ABD), (8–9) ips and cont leg abduction (ABD), (10) static flexion, (11–12) ips and cont leg flexion	3 reps, dynamic and 5 s-static exercise **% MVIC sEMG**	**Single plane:** **GMax: Ips ER:** 46.5 (56.6), **UT: Con ABD:** 66.2 (70.7), **ADelt: Ips ABD:** 45.6 (50.1) **Dynamic:** **LT: PB:** 65.9 (70.7), **IS: PB:** 49.9 (54.4)
[52]	UE, Tr	BB, TB, PM, LD, RA, ES	CKC: (1) chin-ups, (2) lat pull-downs	5 reps, 2 s/rep **normalized sEMG**	**Chin-ups: concentric: BB:** 0.8 (1.1), **LD:** 0.7 (1.1), **ES:** 0.5 (0.8) **Lat pull-down: eccentric: RA:** 0.4 (0.7)
[51]	LE, Tr	VM, VL, RF, BF, ST, GMed, GMax, ES	CKC: forward lunge with dumbbell and elastic bands	3 reps, 10 RM, slow (3 s) and fast (ballistic), 3 loads: 33%, 66%, 100% RM **% MVIC sEMG**	**Elastic:** 84°: **VL:** 105.0 (110.0) and **GMax:** 61.0 (66.0), 54°: **RES:** 51.0 (53.0), 84°: **LES:** 53.0 (59.0), 64°: **GMed:** 62.0 (64.0), 34°: **BF:** 45.0 (48.0), 24°: **ST:** 35.0 (37.0) **Dumbbell:** 84°: **VM:** 113.0 (119.0) and **RF:** 81.0 (86.0)
[59]	LE, Tr	AL, GMax, GMed, RA, EO, ES	(1) land walking (LW), (2) water walking (WW), (3) deep water running (DWR)	self-paced slow, moderate, fast speed, 8 s, 2 reps **% MVIC sEMG**	**DWR:** **Slow: GMax:** 15.2 (8.8), **GMed:** 14.3 (5.6), **AL:** 7.3 (5.9). **Moderate: ES:** 14.8 (10.5), **GMax:** 14.6 (8.4), **AL:** 10.6 (7.4), **EO:** 7.9 (3.8), **RA:** 3.7 (2.3). **Fast: GMax:** 19.1 (12.4), **GMed:** 18.7 (8.0), **AL:** 14.0 (7.5), EO: 10.4 (4.8) **WW: Fast: ES:** 16.6 (8.5)

Region, based on included muscles and dominant side; LE, lower extremity; UE, upper extremity; Tr, trunk; muscle activation (surface electromyogram (sEMG)), mean (standard deviation); MIE, maximal isometric effort; normalized sEMG value in % MVIC, low (≤20% MVIC), moderate (21~40%), high (41~60%), and very high (>60%); BB, biceps brachii; TB, triceps brachii; ADelt, anterior deltoid; MDelt, middle deltoid; PDelt, posterior deltoid; Trap, trapezius; UT, upper trapezius; MT, middle trapezius; LT, lower trapezius; TMaj, teres major; SS, supraspinatus; IS, infraspinatus; LD, latissimus dorsi; SA, serratus anterior; USSC, upper subscapularis; PM, pectoralis major; ES, erector spinae; RA, rectus abdominus; EO, external oblique; TrA, transverse abdominus; IO, internal oblique; ICTL, iliocostalis lumborum pars thoracic; LTh, longissimus thoracic; MF, multifidus; GMax, gluteus maximus; GMed, gluteus medius; RGMed, right GMed; LGMed, left GMed; AL, adductor longus; QF, quadratus femoris; RF, rectus femoris; VM, vastus medialis; VL, vastus lateralis; BF, biceps femoris; ST, semitendinous; SM, semimembranous; TFL, tensor fascia latae; TA, tibialis anterior; PL, peroneus longus; SOL, soleus; GT, gastrocnemius; MGT, medial GT; LGT, lateral GT.

## Data Availability

All data are presented in the manuscript.

## Abbreviations

UE: upper extremity, LE; lower extremity, Tr; trunk, BB; biceps brachii, TB: triceps brachii: ADelt: anterior deltoid, MDelt; middle deltoid, PDelt; posterior deltoid, SS; supraspinatus, IF; infraspinatus, UT; upper trapezius, MT; middle trapezius, LT; lower trapezius, TMaj; teres major, SA; serratus anterior, LD; latissimus dorsi, USSS; upper subscapularis, PM; pectoralis major, GMax; gluteus maximus, GMed; gluteus medius, RF; rectus femoris, VL; vastus lateralis, VM; vastus medialis, AL; adductor longus, TFL; tensor fascia latae, BF; biceps femoris, SM; semimembranous, ST; semitendinous, TA; tibialis anterior, PL; peroneus longus, MGT; medial gastrocnemius, LGT; lateral gastrocnemius, SOL; soleus, RA; rectus abdominus, EO; external oblique, IO; internal oblique, TrA; transverses abdominus, ES; erector spinae, MF; multifidus, ICLT; iliocostalis lumborum pars thoracic, LTh; longissimus thoracic.
